# Analysis of the Placental Tissue Transcriptome of Normal and Preeclampsia Complicated Pregnancies

**Published:** 2014

**Authors:** E. A. Trifonova, T. V. Gabidulina, N. I. Ershov, V. N. Serebrova, A. Yu. Vorozhishcheva, V. A. Stepanov

**Affiliations:** Research Institute of Medical Genetics, Siberian Branch, Russian Academy of Medical Sciences, Nab. Ushayky 10, 634050, Tomsk, Russia; Siberian State Medical University, Ministry of Health of the Russian Federation, Moskovsky Trakt, 2, 634050, Tomsk, Russia; Institute of Cytology and Genetics, Siberian Branch, Russian Academy of Sciences, Prosp. Lavrentieva 10, 630090, Novosibirsk, Russia; City Clinical Hospital № 1, Ul. Khitarova, 32, 654000, Novokuznetsk, Russia; Tomsk State University, Lenina Avenue, 36, 634050, Tomsk, Russia

**Keywords:** microarrays, placenta, genome-wide analysis, preeclampsia, transcriptome, gene expression

## Abstract

Preeclampsia is one of the most severe gestational complications which is one of the leading causes of maternal and perinatal morbidity and mortality. A growth in the incidence of severe and combined forms of the pathology has been observed in recent years. According to modern concepts, inadequate cytotrophoblast invasion into the spiral arteries of the uterus and development of the ischemia-reperfusion syndrome in the placental tissue play the leading role in the development of preeclampsia, which is characterized by multipleorgan failure. In this regard, our work was aimed at studying the patterns of placental tissue transcriptome that are specific to females with PE and with physiological pregnancy, as well as identifying the potential promising biomarkers and molecular mechanisms of this pathology. We have identified 63 genes whose expression proved to differ significantly in the placental tissue of females with PE and with physiological pregnancy. A cluster of differentially expressed genes (DEG) whose expression level is increased in patients with preeclampsia includes not only the known candidate genes that have been identified in many other genome-wide studies (e.g., *LEP*,* BHLHB2*, *SIGLEC6*, *RDH13*, *BCL6*), but also new genes (*ANKRD37*, *SYDE1*, *CYBA*, *ITGB2*, etc.), which can be considered as new biological markers of preeclampsia and are of further interest. The results of a functional annotation of DEG show that the development of preeclampsia may be related to a stress response, immune processes, the regulation of cell-cell interactions, intracellular signaling cascades, etc. In addition, the features of the differential gene expression depending on preeclampsia severity were revealed. We have found evidence of the important role of the molecular mechanisms responsible for the failure of immunological tolerance and initiation of the pro-inflammatory cascade in the development of severe preeclampsia. The results obtained elaborate the concept of the pathophysiology of preeclampsia and contain the information necessary to work out measures for targeted therapy of this disease. ;

## INTRODUCTION

The numerous genome-wide association studies (GWAS) conducted so far have provided valuable information on the genetic architecture of multifactorial diseases (MFDs) and revealed hundreds of the risk alleles of single-nucleotide polymorphisms (SNPs) associated with many phenotypes. However, they explain only a relatively small part of the inheritance of complex traits and have only a very mild impact on the phenotype of associated variants [1]. These results raise the missing heritability problem, which is being intensively discussed today. Another limitation of the GWAS effectiveness related to studies of the hereditary component of predisposition to MFDs is associated with the use of tagSNP. The risk alleles identified in GWAS typically do not belong to the “causal” ones, but are in linkage disequilibrium (LD) with functionally significant variant alleles [2]; therefore, the biological interpretation of GWAS results is a serious problem.

The current approaches to the identification of the “causal” allelic variants linked to the polymorphisms detected in GWAS are based on the analysis of the coding or transcribed genomic regions [2-4]. However, the vast majority of SNPs identified in GWAS are located in the non-transcribed regions. They are not linked to variants located in exons, and the mechanism of their action is apparently associated with the regulation of gene expression [5, 6]. Therefore, post-genomic methods (which readily provide information on almost all the components coordinating the basic functions of genes, RN A, and proteins at different hierarchical levels) become especially relevant in studying the genetic architecture and molecular mechanisms of MFDs. One such approach, namely the high-performance measurement of gene expression using microarray technology, was used in the present work to characterize the transcriptome patterns in normal pregnancy and preeclampsia (PE), one of the most severe gestational complications.

Preeclampsia, which is associated with the multiple organ dysfunction syndrome, is a specific syndrome that occurs after the 20th week of pregnancy and is characterized by hypertension and proteinuria. PE is diagnosed in 70% of hypertensive disorders in pregnancy, and an increase in the incidence rate of severe and combined forms of this disease has been observed in recent years [7]. Despite the large number of theories related to etiopathogenesis (neurogenic, hormonal, placental, immunological, genetic, etc.), numerous studies of the mechanisms of development of this disease, and the emergence of new therapies, PE remains a leading cause of maternal and perinatal morbidity and mortality. The disease is responsible for up to 70% of stillbirths and miscarriages; the risk of perinatal losses increases almost fivefold in PE [7, 8].

According to the modern concepts, the etiopathogenesis of preeclampsia is closely related to inadequate cytotrophoblast invasion in the uterine spiral arteries and development of the ischemia-reperfusion syndrome, which induces oxidative stress and systemic inflammation [9, 10]. Etiological factors and the mechanisms of this disorder remain unclear and require close attention. In order to identify the likely candidate biomarkers of PE and to study the molecular mechanisms of gestational complications, we analyzed the patterns of the placental transcriptome that are specific to PE and physiological pregnancy, since the placental tissue obviously plays the key role in the development of PE. The strategy of using microarrays in this context seems to be reasonable and powerful enough, as it allows one to thoroughly investigate the possible changes in gene expression associated with the pathophysiology of preeclampsia at the transcriptome level.

## EXPERIMENTAL

**Characteristics of the examined groups**

A total of 10 patients with PE and 11 patients with physiological pregnancy (the control group) were examined (*Table 1*). The questionnaire included demographic information (ethnicity) and anthropometric parameters (height, weight), lifestyle (smoking habit, psychoactive substance abuse), as well as information about the somatic and obstetric-gynecological history. PE was diagnosed based on leading clinical symptoms of various severity, such as proteinuria, edema, hypertension (systolic blood pressure above 140 mm Hg, diastolic blood pressure above 90 mm Hg) according to the 10th revision of the International Classification of Diseases. PE severity was evaluated according to the criteria of the clinical protocol 2012 “Hypertension during Pregnancy. Preeclampsia. Eclampsia” [11].

**Table 1:** Characteristics of the examined groups

Parameters	PE,N = 10	Control group N = 11	p-value*
Mean age, years	26 ± 2	28 ± 3	0.241
Mean weight, kg	60 ± 7	62 ± 6	0.324
Body mass index, BMI	23 ± 4	23 ± 3	0.832
The mean maximum systolic blood pressure, mm Hg	162 ± 19	121 ± 3	0.0001
The mean maximum diastolic blood pressure, mm Hg	104 ± 13	80 ± 4	0.0001
Delivery time, weeks	38 ± 1	40 ± 2	0.009
Birth weight, g	2783 ± 560	3549 ± 345	0.004
Length at birth, cm	50 ± 4	53 ± 2	0.021
Premature birth, %	50	0	0.012
Chronic diseases, %	60	50	0.575

* The significance level was determined by comparing the groups using the Mann-Whitney test or Fisher’s exact test.

The group of PE patients was heterogeneous both in terms of severity (the study included six patients with moderate and four patients with severe PE) and the presence of prior diseases and comorbidities. Four patients were diagnosed with PE in the absence of background diseases; in others the gestational complications developed against the backdrop of extragenital diseases including hypo-/hypertensive type neurocirculatory dystonia, chronic pyelonephritis, chronic cholecystitis, and chronic arterial hypertension. Six females in the control group were also diagnosed with both chronic pyelonephritis and chronic cholecystitis. The age of the gravidas ranged from 18 to 33 years in both groups; the groups were comparable in terms of the average age. Statistically significant differences in the height and weight of infants between the control group and the group of patients were found. The groups also differed in terms of arterial blood pressure and time of birth.

**Collection of placental samples**

**Fig. 1:**
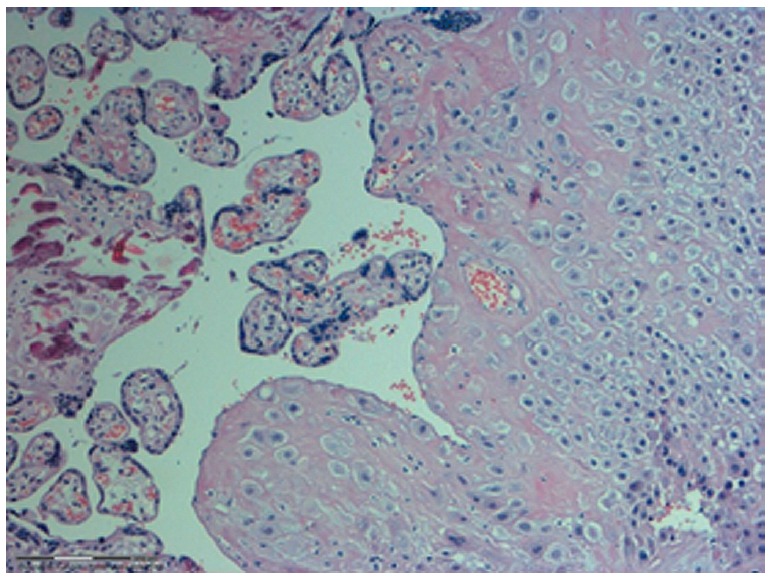
Micrograph of one of the studied placental biopsy samples. Hematoxylin- and eosin-stained.

We examined the distal (maternal) portion of the placenta. The tissues were sampled immediately after delivery (sample ischemia time did not exceed 10 min). Placental tissue samples were taken from the central areas close to the umbilical cord at a placental depth of 0.5 cm. The samples were collected from macroscopically normal sections of the placenta (without hemorrhage, calcification, necrosis, or fibrin deposition) without intervening large vessels, washed with saline to remove the residual maternal blood and amniotic fluid, immediately immersed in RN Alater (Ambion, UK), and transferred to be stored at –80°C until the RN A iso lation procedure. A histological examination revealed chorionic villi and decidua tissue with fibrinoid necrosis foci and small calcifications in all biopsy specimens (*Fig. 1*).

**RNA Isolation**

Tissue samples (100–200 mg) were homogenized using TissueLyser (Qiagen) in Trizol; RN A was then isolated using the standard protocol. The concentration of total RN A was determined using Nanodrop ND-1000 based on absorbance at 260 nm in water. The quality of the samples was monitored using an Agilent 2100 Bioanalyzer capillary electrophoresis system (Agilent Technologies Inc., Palo Alto, USA) and spectrophotometric scanning.

**Microarray analysis**

A genome-wide profile of gene expression in the placental tissue was determined using hybridization on HT-12 BeadChip microarrays (Illumina, USA) containing information about more than 48,000 transcripts. After hybridization, the microarrays were scanned on an Illumina BeadArray Reader device. The raw data were converted into mean values of the signal intensity for each sample (Sample Probe Profile) using the BeadStudio v3 software package (Illumina).

**Bioinformatic analysis**

The data were analyzed in an R software environment using the limma program package [12]. Nonparametric background correction followed by quantile normalization (neqc function) was performed for the entire data set. The specimens that were identified in all the samples of at least one of the experimental groups (detection p-value < 0.01) were further considered. A differential expression analysis was performed using multiple linear regression and moderated t-statistics [12], including the assessment of the quality weights of microarray reading [13] and Benjamin-Hochberg multiple testing correction (FDR) procedure. A 1.5-fold (or greater) change in the level of gene expression (FC – fold change) was considered to be significant at the adjusted significance level of p ≤ 0.1. Functional annotation and functional cluster analysis of the groups of differentially expressed genes (DEGs) were performed using the DAVID (Database for Annotation, Visualization and Integrated Discovery) web-based tool with the standard values of clustering parameters and enrichment score EASE ≤ 0.01 [14]. Construction of gene networks was performed using the STR ING 9.0 program (Search Tool for the Retrieval of Interacting Genes) [15].

This study was approved by the Ethics Committee at the Research Institute of Medical Genetics, Siberian Branch of the Russian Academy of Medical Sciences.

## RESULTS

Our analysis identified 63 genes with significantly different expressions (FDR < 0.1; FC ≥ 1.5) in the placental tissue of females with PE and physiological pregnancy (50 DEGs with an increased expression level and 13 DEGs with a decreased expression level). The DEG cluster, whose expression was increased in PE, includes not only known candidate genes that have previously been identified in many genome-wide studies of the expression profiles of the placental genes in preeclampsia (e.g., *LEP*, *BHLHB2*, *SIGLEC6*, *RDH13*, *BCL6*), but also new potential candidate genes (*CORO2A, SYDE1, PLIN2, CEBPA, HK2, NDRG1, ERRFI1, EFNB1, GFOD2, NCOR2, HMHA1, HERPUD1, KIF2A*), whose association with the development of PE has been established either in a few studies [16-21], or was done so for the first time in our work. The products of some of these genes, based on current knowledge on their functional features, can be involved in the etiopathogenesis of PE.

**Fig. 2:**
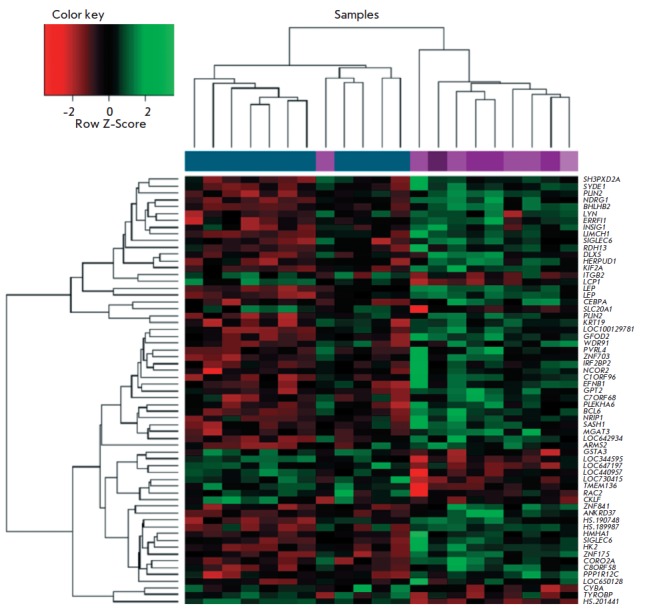
Heatmap of DEGs (FDR < 0.1; FC ≤ 1.5). Each column represents a sample; each row represents DEG. Samples from PE patients are shown in pink; samples from the females of the control group are shown in blue. The color scale of the heatmap indicates the deviation of the normalized expression level in the cell from the mean value for the row

*Figure 2* shows the heat map with the results of a hierarchical clustering of females according to the expression level of 63 DEGs. It can be seen that all PE patients but one fall into one cluster, while females with physiological pregnancy fall into the other one. One PE sample was assigned to the control group probably due to the significant interindividual variability of the transcription levels of the placental tissue genes. A similar phenomenon was observed in several human cell lines: in particular, in the cell lines of the hepatocyte transcriptome [22, 23].

**Table 2:** Threshold cycle values and the calculated number of DNA copies in the model samples after real-time PCR

N^o^	Change in expression level	FC	FDR	Gene	Chromosome	Gene product	Primary functions*
1	↑	1.054	0.0007	LEP	7	Leptin	The gene encodes the protein which is secreted by adipocytes and plays an important role in the regulation of food intake and / or energy consumption to maintain the constancy of the adipose mass. Leptin is also involved in the regulation of immune and inflammatory reactions, and processes of hematopoiesis, stimulation of angiogenesis and inhibition of apoptosis.
2	↓	3.84	0.0001	HS.201441	13	Long non-coding RN A 284	According to the analysis of EST-libraries, tissuespecific expression of HS.201441 gene is mainly observed in the placenta and ovaries.
3	↑	2.95	0.0000	BHLHE40	3	Transcription factor including the helix-loop-helix domain	Transcription factor modulating chondrogenesis within the cAMP-signaling pathway. It is involved in the control of cell differentiation.
4	↑	2.93	0.0031	ANKRD37	4	Ankyrin 37 repeat domain	The encoded protein contains four ankyrin repeats, which are mediators of protein-protein interactions and are involved in the regulation of the functioning of key transcription factors, such as NFκB and TP53. In addition, there is evidence of the important role of ANKRD37 in the cellular response to hypoxia [24].
5	↑	2.91	0.0067	SIGLEC6	19	Immunoglobulin-like lectin 6, which binds to the sialic acid	Immunoglobulin-like lectins that bind to sialic acids are a family of type 1 membrane proteins which recognize and bind sialylated glycans. SIGLEC 6 interacts with the α-2,6-bound sialic acid of cellular membranes of immune cells and regulates cell adhesion, thus participating in the immune response. Furthermore, SIGLEC 6 is a ligand of leptin [25].
6	↑	2.64	0.0006	ZNF175	19	Zinc finger protein 175	Negative regulation of the expression of various chemokine receptors.
7	↑	2.43	0.0031	CCSAP (C1orf96)	1	Protein localized in centriole, cilia and cleavage spindle (open reading frame 96, chromosome 1)	Presumably plays a role in embryonic development
8	↑	2.43	0.0050	GPT2	16	Alanine aminotransferase 2; glutamate pyruvate transaminase	Catalyzes the reversible transamination between alanine and 2-oxoglutarate to form pyruvate and glutamate participating in amino acid metabolism and gluconeogenesis.
9	↑	2.39	0.0050	RDH13	19	Retinol dehydrogenase 13	Catalyzes oxidation and reduction of retinoids and participates in protecting mitochondria from oxidative stress.
10	↑	2.36	0.0006	BCL6	3	Transcription factor – zinc finger protein 51	Contains N-terminal POZ/BTB-domain and is a sequence-specific transcription repressor. Participates in modulation of STAT-dependent IL-4-induced immune response involving B cells, antibody formation, and lymphangiogenesis.
11	↑	2.33	0.0056	PLIN2	9	Perilipin 2, lipid droplet protein	Perilipin function in basic cells is to stabilize the storage of neutral lipids. Its functions are related to providing PKA-activated lipolysis in activated cells.
12	↑	2.31	0.0174	NRIP1	21	Nuclear factor RIP140	Modulates the transcriptional activity of steroid receptors such as NR 3C1, NR 3C2, and ESR1 in the nucleus. Can act as a transcriptional activator or repressor, depending on the transcription factors it interacts with [26].
13	↑	2.18	0.0407	HILPDA (C7orf68)	7	Hypoxia-induced protein 2 (open reading frame 68, chromosome 7)	The gene encodes hypoxia-induced factor 2, which promotes the intracellular accumulation of lipids, stimulates the expression of cytokines, including IL- 6, MIF, and VEGFA, and enhances growth and proliferation of cells.
14	↑	2.18	0.0006	SYDE1	19	GTPase-activating protein, homolog 1	This protein belongs to the Rho GTPase family, which plays an important role in cell proliferation, apoptosis, gene expression, and regulation of intracellular actin dynamics.
15	↑	2.06	0.0050	CORO2A	9	Coronin 2A	Belongs to actin-binding protein family, which performs important functions related to cell motility, membrane transport, and cell-cell signal transduction. It was shown that coronin 1A mediates Toll-like receptors and is involved in the inflammatory response [27].

* Information from the GeneCards database (http://www.genecards.org/) including addendum.

*Table 2* shows the data related to the most significant DEGs (FC > 2, FDR < 0.01). The presence of several genes whose products are involved in the transcriptional regulation (BHLHB2, ZNF175, ANKRD37, BCL6) in this list, as well as a significant increase in the expression level of the *LEP *gene and its receptor gene *SIGLEC6 *during the development of PE, is of special interest.

**Fig. 3:**
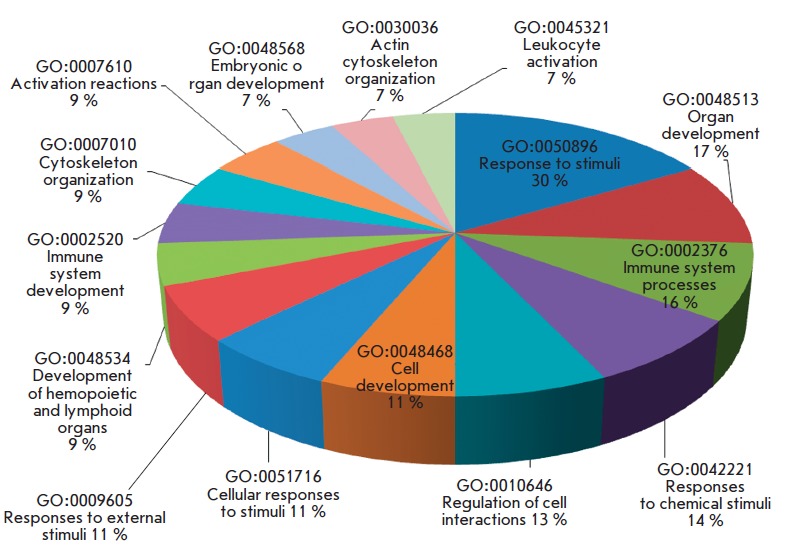
Main biological processes involving DEGs, which are associated with preeclampsia (p < 0.05). The percentage indicates the proportion of identified DEGs associated with this process.

We analyzed DEG using the DAVID online resource to study the biological processes associated with the development of PE (*Fig. 3*). The major categories of molecular functions of the protein products of these genes include responding to various stimuli, immune processes, regulation of cell communication, intracellular signaling cascades, etc. The analysis of metabolic pathways including DEGs has shown that cytotoxicity pathways associated with NK-cells, transendothelial migration of leukocytes, and signaling pathways mediated by GTPase activators are probably involved in the molecular mechanisms of PE.

**Fig. 4:**
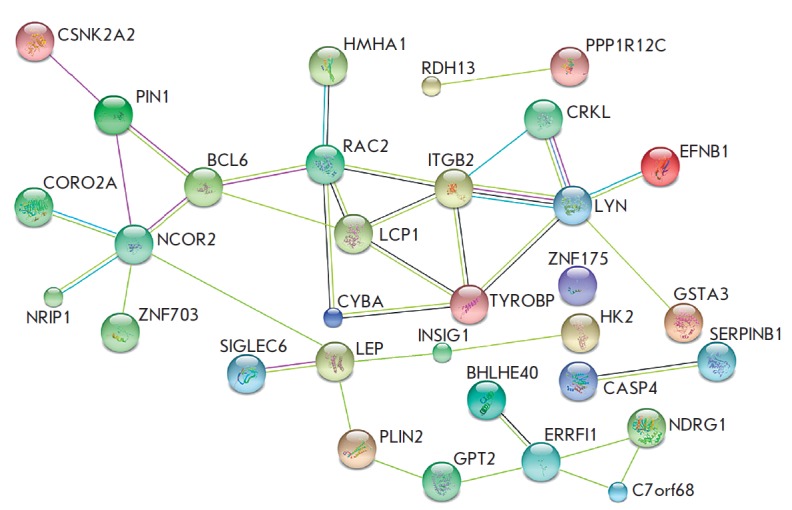
Protein–protein interactions between DEG products. The proteins are shown as circles; the color line between these circles indicates the evidential category of protein–protein interaction: yellow – literature data (“text mining”), black –according to the analysis of gene coexpression, purple – the experimental results, blue – evidence from the databases, pink – cumulative evidence

Protein-protein interactions of DEG products were analyzed to identify the possible relationships with DEGs (*Fig. 4*). The associations in the constructed network are mainly based on “text mining” (mentioned in the abstract of one article). The coexpression cluster that includes the *RAC2*, *CYBA*, *TYROBP*, *HMHA1*,* ITGB2*, *LYN *and *LCP1 *genes should be mentioned. In addition, LEP and its receptor SIGLEC 6 and ephrin with its kinase LYN are of certain interest.

Our study also revealed features of differential gene expression depending on PE severity (*Table 3*). A total of eight DEGs were identified (FDR < 0.1; FC ≤ 1.5), whose expression levels were significantly different in moderate and severe forms of the disease. In our opinion, *HSPA1A* encoding the highly conserved heat shock protein 70 (HSP70) and *BAG3 *encoding Bis, a Bcl-2 binding protein, are of the greatest interest. The main function of the Bis protein is inhibiting the chaperone activity of the HSP70/HSC70 complex.

**Table 3:** List of differentially expressed genes (FDR < 0.1; FC ≤= 1.5) in="" moderate="" to="" severe="" preeclampsia=""

Gene	Change in gene expression level	FC	FDR	Gene product
HSPA1A	↑	6.44	0.079549	Heat shock protein 70, HSP70-1A
BAG3	↑	2.14	0.073131	Bcl-2-associated athanogene 3
SNHG8	↑	1.78	0.04105	Small nucleolar RN A 8
LOC729660	↓	2.63	0.010437	No data
LOC728457	↓	2.43	0.010437	No data
APOC1	↓	2.28	0.04433	Apolipoprotein C1
LOC401357	↓	2.27	0.010399	No data
LOC100128326	↓	1.92	0.079549	No data

**Fig. 5:**
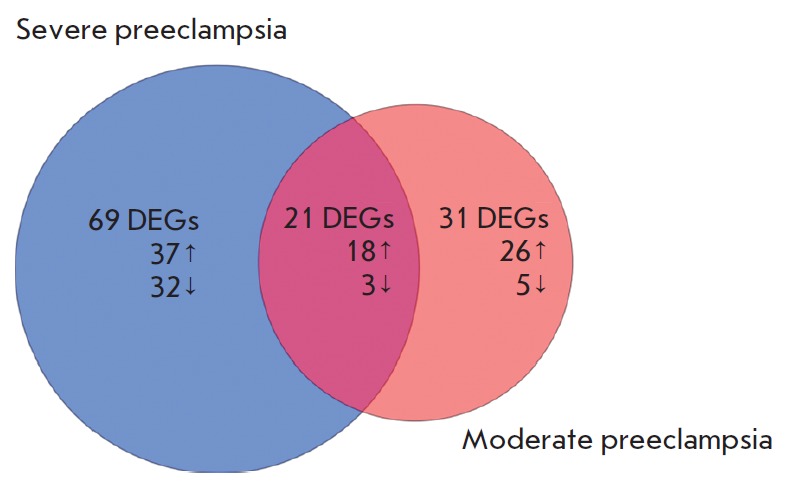
Venn diagram showing the results of the gene expression profiling in moderate and severe preeclampsia and in physiological pregnancy. DEGs – genes that are differently expressed in females with preeclampsia and physiological pregnancy (control group). The arrow shows the increase (↑) or decrease (↓) in gene expression.

**Table 4:** Main biological processes that involve differentially expressed genes characteristic of severe preeclampsia

Categories of biological processes	Gene	p*
Processing and presentation of peptides or polysaccharide antigens via class II MHC molecules (GO:0002504)	HLA-DPA1, CD74, HLA-DMA, HLA-DRA	0.0421
Processing and presentation of exogenous peptide antigens (GO:0002478)	HLA-DMA, CD74, HLA-DRA	0.0453
Chaperone-mediated protein folding (GO:0051085)	ERO1L, HLA-DMA, CD74	0.0467
De novo post-translational protein folding (GO:0051084)	ERO1L, HLA-DMA, CD74	0.0478
Reactions of unfolded protein molecules (GO:0006986)	ERO1L, HSPH1,HSPA1A, HERPUD1	0.0489

* Significance level including Benjamin-Hochberg multiple testing correction, which characterized the accuracy of the assignment of this set of genes to a certain biological process.

A comparative analysis of gene expression profiles in the placental tissue of females with moderate PE and in the control group revealed 56 transcripts of 52 genes, whose transcription levels differ significantly in these populations. Changes in the expression profile were more pronounced in severe PE: a significant increase in the expression of 55 genes and a decrease in the expression of 35 genes compared to physiological pregnancy were observed (*Fig. 5*). It should be noted that, along with a small amount of common genes (21 genes) that were differentially expressed in both severe and moderate PE, more than 60 DEGs were specific only to the severe form of the pathology. The results of a functional annotation of these genes in the DAVID web-resource point to a number of biological processes that are statistically significantly associated with the development of severe PE, such as processing and presentation of peptide or polysaccharide antigens and protein folding (*Table 4*). An analysis of the metabolic pathways that involve these genes also demonstrates the important role of the mechanisms of processing and presentation of antigens in the molecular pathogenesis of severe PE (according to the KEGG and BIOCART A databases).

## DISCUSSION

The placenta is the key in understanding the physiological processes associated with pregnancy. It is important to characterize the genes essential for placental function to understand the mechanisms underlying normal and pathological gestation. The results of this work show that the identified DEGs belong to several biological processes associated with immune responses, cell-cell interactions, and responses to various stimuli. It should be noted that the analysis performed using the module for functional annotation clustering of the DAVID bioinformatical resource made it possible to identify 16 clusters. However, only one of them had an enrichment score of over 2. This cluster includes five genes (*KRT19*, *RAC2*, *LIMCH1*, *BCL6*, *LCP1*) involved in the biological processes related to the organization of the actin cytoskeleton (GO: 0030036; GO: 0030029; GO: 0007010). Studying the functional role of the actin cytoskeleton is one of the important trends in the study of cellular signaling mechanisms. Numerous experimental data published over the past few years provide evidence of the fact that actin is involved in the regulation of gene expression and mediates it by participating in transcription elongation, assembly of the preinitiation complex, mRN A maturation and export, chromatin reorganization, and other processes [28, 29]. In this context, the increase in the expression level of the *CORO2A *gene seems interesting. The product of this gene, coronin 2A, belongs to the family of actinbinding proteins and mediates the Coro2A/actin-dependent mechanism of derepression of the inflammatory response genes [27].

We found no association between the development of PE and such canonical pathways as abnormal apoptosis and angiogenesis as described in several papers [16, 19, 30, 31]. This is probably attributable to the interethnic variability of the gene expression profiles in the placental tissue due to the population differentiation of the regulatory regions of the genome, or due to the different criteria (population size, delivery time, severity of the disease, etc.) used in the formation of the examined groups. The different placental localization of the biopsy samples used in individual studies of the transcriptome in PE is another factor that apparently affects the occurrence of these contradictions. Thus, highthroughput sequencing (RN A-Seq) revealed significant differences in the gene expression profiles in the amnion, chorion, and decidua of the human placenta [32]. Similar findings were previously arrived at when performing a microarray analysis of the transcriptome patterns in different portions of the placenta [33].

Despite the aforementioned differences in the results of the DEG functional annotation, it remains of interest that changes in the expression levels of some DEGs identified in our work were also described in other studies (*Table 5*).

**Table 5:** Differentially expressed genes identified in this study whose association with preeclampsia has been previously shown in studies focused on the placental tissue transcriptome

N^o^	Gene	Gene product	FC	Significance level	Ethnic populations	Reference
1	LEP	Leptin	10.94	< 0.0001*	Japanese	[30]
			8.58	0.036*	Chinese	[34]
			40.11	1.35 × 10^-9^	Caucasian	[16]
			5.52	0.0020	Caucasian Afro-American Mongoloid Spaniard	[18]
			108.9	< 0.0001	Caucasian	[35]
			4.4	< 0.0001	Korean	[36]
			≥ 1.5	< 0.05	Chinese	[37]
			11.79	< 0.01*	American	[38]
2	BCL6	Transcription factor – zinc finger protein 51	1.78	0.0154	Caucasian Afro-American Mongoloid Spaniard	[18]
			2.02	0.0024	Japanese	[30]
			2.24	3.58 × 10^-5^	Caucasian	[16]
			2.60	< 0.01*	American	[38]
			≥ 1.5	< 0.05	Chinese	[37]
3	SIGLEC6	Immunoglobulin-like lectin 6, which binds to sialic acid	-	0.02*	American	[31]
			2.13	0.001	Caucasian	[16]
			2.73	< 0.01*	American	[38]
			≥ 1.5	< 0.05	Chinese	[37]
			4.5	0.019	Caucasian	[35]
4	RDH13	Retinol dehydrogenase13	-	3.86 × 10^-8^*	American	[31]
			1.91	< 0.01*	American	[38]
			≥ 1.5	< 0.05	Chinese	[37]
5	NDRG1	Cytoplasmic protein belonging to the hydrolase superfamily	2.02	0.0001	Japanese	[30]
			2.67	1.12 × 10^-5^	Caucasian	[16]
6	BHLHE40	Transcription factor with helix-loophelix domain	1.95	0.0004	Japanese	[30]
			3.08	2.18 × 10^-5^	Caucasian	[16]
7	KRT19	Keratin 19	1.75	0.0071	Japanese	[30]
			2.28	1.59 × 10^-5^	Caucasian	[16]
8	GPT2	Alanine aminotransferase 2	2.45	3.70 × 10^-5^	Caucasian	[16]
9	PPP1R12C	12A regulatory subunit of phosphatase 1	-	2.16 × 10^-8^*	American	[31]
10	CEBPA	CC AAT/enhancer-binding protein α	-	2.52 × 10^-8^*	American	[31]
11	HK2	Type 2 hexokinase	3.90	3.87 × 10^-6^	Caucasian	[16]
12	HMHA1	Minor histocompatibility antigen HA1	-	1.23 × 10^-8^*	American	[31]
13	PVRL4	Nectin 4	aaaa	aaaa	Caucasian	[16]
14	SASH1	SAM- and SH3-domain-containing protein 1	2.54	1.22 × 10^-7^	Caucasian	[16]
15	SH3PXD2A	SH3- and PX-domain-containing protein 2A	≥ 1.5	< 0.05	Chinese	[37]
16	SYDE1	GTPase activating protein, homolog 1	1.55	< 0.01*	American	[38]

* Significance level including multiple testing correction.

Thus, a significant increase in *LEP *gene expression in preeclampsia was observed in almost all genomewide studies of gene expression profiles in the placental tissue. Leptin, the product of this gene, is one of the new serum markers of PE. It is known that leptin belongs to adipocyte-specific cytokines that regulate energy metabolism and are involved in various metabolic and neuroendocrine processes [39]. The studied group of PE patients did not differ significantly from the control group of patients in terms of weight and body mass index. None of the patients had abnormal weight gain during pregnancy, and therefore it can be assumed that the contribution of leptin to the development of PE is determined by other functions of this protein. Placental leptin is known to be involved in providing the flow of nutrients to the fetoplacental complex and to induce the trophoblast proliferation by inhibiting apoptosis [40, 41]. Thus, an increase in the leptin level in the placenta may be a compensatory mechanism directed against endothelial dysfunction, which is observed in PE. Meanwhile, it was shown that leptin is involved in the activation of the sympathoadrenal system, which contributes to arterial hypertension, the main symptom of PE [42]. In addition, an important immunomodulatory function of leptin was found, which may also contribute to the development of pregnancy failure [43]. Despite the intensive studies devoted to *LEP *gene expression, only some works have focused on the analysis of the hereditary variability of this gene and its role in changing the transcription level and the structure of susceptibility to pregnancy failure. It was shown that carriers of the AA genotype of the rs2167270 locus (G19A) located in the promoter region of the *LEP *gene have elevated levels of expression of this gene in the blood, as well as the risk of PE and hypertension [44]. Association between another polymorphism (G2548A) localized at the *LEP *gene promoter with gestational diabetes [45] was found in the Czech population. Along with this, several studies [37, 46, 47] have revealed significant hypomethylation of this locus, as well as dysregulation of the placental epigenome during the development of PE.

The increase in the expression of the long non-coding RN A 284 gene in the placental tissue of PE patients, which was observed in our work, is of particular interest in the context of the role of epigenetic dysregulation in the formation of this pathology. It has recently been shown that long ncRN As perform vital regulatory functions in cells. In particular, it is assumed that they can function as a module scaffold in the specific, highly ordered organization of ribonucleoprotein complexes and induction of epigenetic changes in these loci. Some long ncRN As can bind chromatin using remodeling enzymes and then participate in the local chromatin modification, e.g. in DNA methylation, by initiating or repressing transcription. This RN A class can participate in the binding of transcription factors and inhibiting gene expression [48].

The association between overexpression of the* BAG3 *and *HSPA1A *genes and the development of severe PE seems to be of interest. The protein product of the *BAG3 *gene is known to compete with Hip cochaperone for binding to the ATPase domain of the HSP70/HSC70 complex and thus inhibits the chaperone activity of the heat shock protein 70 (Hsp70), the product of the *HSPA1A *gene, whose expression is significantly elevated in severe PE (more than sixfold as compared to moderate PE and eightfold as compared to the control population). It is known that Hsp70 performs various functions. Improving the resistance of the protein biosynthesis apparatus to damaging influences and chaperone activity are the most significant of them. In addition, there is data indicating that Hsp70 participates in protein transport, conduction of the intracellular signal, and protease-dependent degradation [49]. It should be noted that according to the results of the functional annotation of DEGs, processing and presentation of peptide and polysaccharide antigens and chaperone-mediated protein folding are the principal processes characteristic of severe PE. Since the heat shock protein Hsp70 is capable of forming complexes with non-folded proteins and a wide variety of peptide fragments that are precursors of the antigenic peptides presented on the cell membrane along with other class I and II MHC molecules [50], it is reasonable to assume that the immunological control mechanisms of trophoblast invasion in the uterine wall and the immunological tolerance factors in the mother-fetus system play a key role in the pathogenesis of severe PE. The pathological effect of these factors can lead to gestational complications. Furthermore, many heat shock proteins exhibit immunoregulatory activity, stimulate the maturation of dendritic cells, and induce some proinflammatory cytokines [51]. These properties of these proteins may also contribute to the mechanisms of severe PE.

The statistically significant decrease in the expression of the *APOC1 *gene in severe PE, which was revealed in the present study, is probably due to the development of oxidative stress in the blood vessels of the placenta or the recently discovered immunosuppressive properties of the C1 apolipoprotein encoded by this gene [52]. It was previously shown that the serum of PE patients has a high level of triglyceride-rich lipoproteins, which can promote endothelial dysfunction [53, 54]. Meanwhile, the blood level of E and A1 apolipoproteins is decreased in this pathology [55, 56]. In addition, a protective role of the *APOE *ε2 allele in the Kurd population was demonstrated, which is related to the high antioxidant properties of this allele according to the authors [57]. We failed to find any information about an association between *APOC1 *polymorphisms and gestational complications. However, it was shown that the insertion-deletion polymorphism at –317 position of the promoter region of this gene (rs11568822) is associated with Alzheimer’s disease, while the rs4803770 marker is associated with the coronary heart disease [58, 59]. Since the *APOC1 *gene localizes in the same cluster as the *APOE *gene, it is assumed that these associations are due to the strong linkage disequilibrium between these genes [60]. However, we found no statistically significant changes in the expression level of the *APOE* gene. Therefore, it is reasonable to consider the *APOC1* gene to be an “independent” new candidate gene for PE. However, this assumption needs confirmation.

Thus, the findings indirectly confirm the immunological hypothesis of the development of severe PE, which postulates the key role of immune competent cells (B lymphocytes, monocytes, dendritic and NK cells) in the pathophysiology of this disease. This theory assumes that the etiopathogenesis of PE is triggered by insufficient trophoblast invasion into maternal spiral arteries, which is associated with a decreased expression of HLA antigens and “aggression” against NK cells. This results in reduced placental perfusion and development of hypoxia at the mother/fetus boundary, which, in turn, triggers the activity of pro-inflammatory cytokines, leading to endothelial dysfunction [61]. B cells may also contribute to the development of preeclampsia by producing anti-adrenoceptor antibodies.

## CONCLUSIONS

The present work is the first Russian genome-wide study of differential gene expression in the placental tissues in normal and complicated pregnancies. The results indicate that some processes can play an important role in the molecular pathogenesis of PE: reactions associated with the immune response, cytoskeleton organization, cell-cell interactions, responses to various stimuli, and chaperone-mediated protein folding. Integration of the results of a functional annotation of DEGs, analysis of network interactions of the proteins encoded by these genes, and the study of the transcriptome of the placental tissue make it possible to identify a number of novel genes that could be associated with PE: *LEP, SIGLEC6, BHLHE40, BCL6, RDH13, HSPH1, HSPA1A, BAG3, KRT19, RAC2, LIMCH1, BCL6 *and *LCP1*.

We have also obtained evidence of a significant contribution of oxidative-stress-increasing expression of the genes of the Hsp70 and Hsp105 heat shock proteins, which are involved in the molecular mechanisms associated with impaired immune tolerance and initiation of the pro-inflammatory cascade, to the development of severe PE. Meanwhile, the observed increase in *BAG3* gene expression is probably due to the compensatory mechanisms or anti-apoptotic properties of the protein encoded by this locus. This assumption is supported by a statistically significant increase in the expression of the Hsp70 and Hsp90 heat shock proteins, heat shock factor 1 (HSF1), and Bcl-2 anti-apoptotic factor in endothelial cells of the placenta of PE patients as compared to females with normotensive pregnancy [62]. Moreover, the analysis of the proteome of placental tissue from females with physiological and complicated pregnancies has revealed the significance of stress-inducible proteins, including Hsp70, in the pathogenesis of PE [63].

The findings may be useful for understanding the molecular mechanisms of PE and searching for new candidate genes and biomarkers for this pathology. In addition, they provide information for the development of targeted therapy for this disease.

## Abbreviations

DEG: differentially expressed genes
MFDs: multifactorial diseases
PE: preeclampsia
GWAS: genome-wide association study
SNP: single nucleotide polymorphism
